# Synthesis and Reactivity of Oligo(ethylene glycol)-Tethered Morita–Baylis–Hillman Dimers in the Formation of Macrocyclic Structures Showing Remarkable Cytotoxicity

**DOI:** 10.3390/ph18040473

**Published:** 2025-03-27

**Authors:** Marco Paolino, Mario Saletti, Jacopo Venditti, Arianna Zacchei, Alessandro Donati, Claudia Bonechi, Germano Giuliani, Stefania Lamponi, Andrea Cappelli

**Affiliations:** Dipartimento di Biotecnologie, Chimica e Farmacia, Università degli Studi di Siena, Via Aldo Moro 2, 53100 Siena, Italyandrea.cappelli@unisi.it (A.C.)

**Keywords:** macrocyclic compounds, crown ethers, paracyclophane derivatives, cytotoxicity, anticancer agents

## Abstract

**Background/Objectives**: Crown ethers have received increasing interest owing to their ability to form stable complexes with cations. This molecular feature has been successfully exploited in the development of biologically relevant ionophores. **Methods**: In order to obtain innovative crown ethers derivatives, a Morita–Baylis–Hillman adduct (MBHA) acetate (**4**) bearing a phenylacetylene moiety was dimerized via the click-chemistry CuAAC reaction with oligo(ethylene glycol) diazide derivatives to build-up a small series of dimeric MBHA derivatives (**5a-d**). These dimeric MBHA derivatives were reacted with n-butylamine to afford tunable macrocyclic crown ether-paracyclophane hybrid architectures (**6a-d**). **Results**: Compounds (*E*,*Z*)-**6a**, (*E*,*E*)-**6a**, **6b-d** showed, in human breast cancer MDA-MB-231 and human melanoma A375 cells, IC_50_ values comparable with those of reference anticancer agent Doxorubicin. **Conclusions**: This exploration approach provides original new macrocyclic architectures potentially useful as anticancer agents.

## 1. Introduction

Crown ethers are macrocyclic oligomers of ethylene oxide that exist in two forms: aliphatic and aromatic [1,2,3]. They were named on the bases of the evidence on the formation of host–guest complexes with metal cations [3,4]. In fact, the unique conformational and chemical characteristics of crown ethers allow them to create an electron-rich cavity, making them selective for alkali, alkaline-earth ions, and even non-metallic species. As a result, in the biological field, crown ethers can play a role in artificial intramembrane channels and enable the translocation of ionic species [2,5,6,7]. For example, a study reported the potential cytotoxicity of hydraphile compounds to bacterial [8] and mammalian cells presumably due to the unregulated ion flux through the plasma membrane [9] and even as discussed by Cai and Arenaz some crown ethers can show antiproliferative activity [10]. These assumptions together with the awareness that some ionophores, such as valinomycin [11,12], have been reported to display antitumor effects, led Marjanovic and co-workers to check the possible antiproliferative/antitumor ability of crown ethers [13]. Their findings support the hypothesis that the powerful antiproliferative activity of several crown ether compounds tested is the outcome of their activity as membrane-active potassium ion transporters, which can promote K^+^ efflux [13].

Then, the cell membrane can be targeted by cationic ionophores (including crown ethers) [14], causing an imbalance in major physiological cations homeostasis and toxic consequences even in normal tissues [15]. Indeed, past studies have shown that these cationic ionophores can cause reversible toxic effects in several species, such as neurobehavioral effects, eye and skin irritation, and testicular atrophy [16]. Because of that the authors believe that crown ethers (or other membrane-active drugs) could be used as alternative anticancer drugs since they should induce different toxicity than conventional drugs and could complement existing therapy also thanks to their demonstrated ability to inhibit the efflux of anticancer drugs through P-glycoprotein (P-gp) [17].

The results of the study mentioned above also indicate that crown ethers show significant inhibition of cancer cell growth that is closely related to the composition of the hydrophilic cavity and the characteristics of the surrounding hydrophobic ring. The most effective compounds proved to be di-*tert*-butyldicyclohexano-18-crown-6, and di-*tert*-butyldibenzo-18-crown-6, which showed significant cytotoxicity. Additionally, these compounds significantly affected the distribution of cell cycle phases, causing an arrest in the G1 phase followed by the induction of apoptosis [18]. The importance of molecular structure, orientation of hydrophobic groups, and distribution of polarizable elements for interaction, presumably, with cell membranes, has been highlighted. Therefore, their results support the hypothesis that crown ethers may inhibit cancer cell growth through the perturbation of potassium ion homeostasis, which, in turn, leads to cell cycle perturbations and apoptosis, but further in vitro and in vivo studies are needed to confirm it and evaluate their potential clinical use [13,18].

For the synthesis of the crown ethers described below, we have applied the concept of “click” chemistry that involves linking small modular units in reactions with specific characteristics like high yield, chemoselectivity and stereospecificity, high thermodynamic driving force, irreversible bond formation, and mild conditions with few or no by-products [19,20]. The Copper(I)-Catalyzed Azide-Alkyne Cycloaddition (CuAAC) is the best-known “click” reaction, and it is commonly used in mechanically interlocked molecule (MIM) synthesis, including the creation of rotaxanes and catenanes [21,22].

On the other hand, the Morita–Baylis–Hillman reaction is essential in organic chemistry as it allows the formation of new C-C bonds, involving a carbon electrophile, an activated alkene, and catalysts such as tertiary amine or phosphine [23]. It results in the formation of an allylic alcohol that can be acetylated to become an excellent starting group. This reaction allows the creation of versatile and chiral products demonstrating the effectiveness of organocatalysis for green chemistry [24,25].

**Figure 1:**
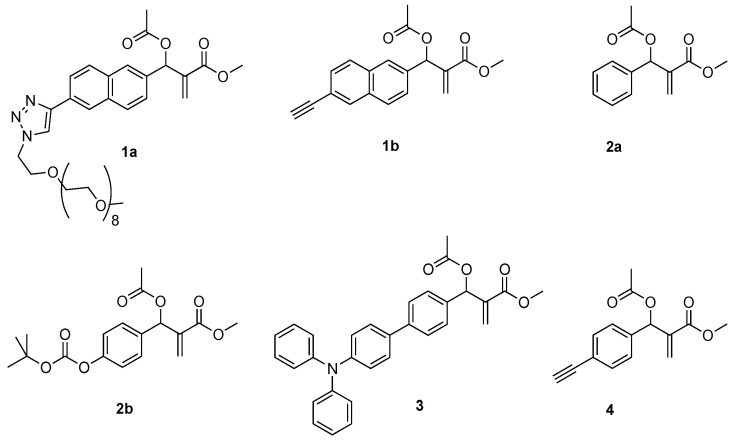
Structure of MBHA derivatives **1a**,**b**, **2a**,**b**, **3**, and **4**.

Several Morita–Baylis–Hillman adduct (MBHA) derivatives were designed and synthesized in our laboratories to react with imidazole, histidine derivatives, n-butylamine, lysine derivatives, and proteins [26,27,28,29,30,31,32,33,34,35], and the reactivity of some MBHA compounds was evaluated in protein models. Very interestingly, the results obtained with a single chain Fv antibody suggested that MBHA derivative **1a** (Figure 1) reacted with the hexahistidine tag producing multi-PEGylated species [28,29].

On the other hand, MBHA derivatives **1b**, **2a**,**b** (Figure 1) were found to react with a lysine residue embedded in a lipophilic pocket of a retinoic acid binding protein [30,36]. Finally, MBHA derivative **3** was found to react with human serum albumin (HSA) producing a significant shift in the emission from the blue to the green-yellow, thus leading to green fluorescent albumin (GFA) derivatives [33]. Since docking studies suggested a possible interaction between the acrylic activated moiety of compound **3** (Figure 1) with a lysine residue located into a lipophilic pocket in the HSA structure [33], the reactivity of MBHA derivative **3** was evaluated with n-butylamine, Nα-acetyl-*L*-lysine methyl ester, and a poly-(*L*-lysine) derivative as models of the lysine amino acid residues [35]. In the reaction of compound **3** with n-butylamine, or with Nα-acetyl-*L*-lysine methyl ester we observed the formation of the corresponding diadduct derivatives along with the monoadduct ones in variable proportions depending on the reaction conditions. In particular, compound **3** showed the tendency to produce diadducts especially in polar solvents systems where stacking interactions between the extended aromatic moieties may play a major role in stabilizing the diadduct structure [35].

**Scheme 1:**
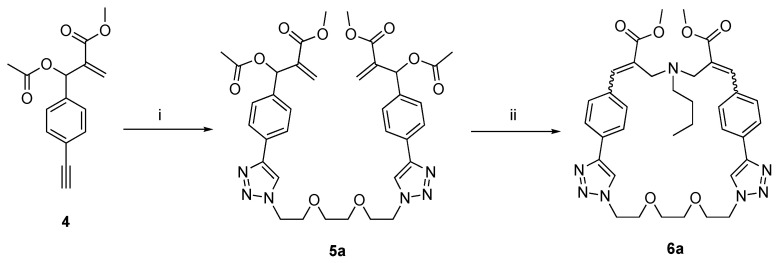
Synthesis of MBHA dimer **5a** and reaction with n-butylamine leading to macrocyclic crown ether-paracyclophane hybrid structures **6a**. Reagents: (i) N_3_-CH_2_CH_2_O-CH_2_CH_2_O-CH_2_CH_2_-N_3_, CuBr(I), DIPEA, CH_3_CN; (ii) CH_3_CH_2_CH_2_CH_2_NH_2_, CHCl_3_.

Very recently, MBHA derivative **4** was used as a synthon in the convergent synthesis of tri(ethylene glycol)-tethered MBHA dimer **5a** (Scheme 1), which was designed to be used in the functionalization of materials containing reactive basic groups such as amino or imidazole moieties [32]. The reactivity of tri(ethylene glycol)-tethered MBHA dimer **5a** was studied with n-butylamine as the simplest model of a lysine residue, and this reaction lead to the formation of macrocyclic crown ether-paracyclophane hybrid structures **6a** that could be modulated by light [32].

In the present work, the tri(ethylene glycol) component of the synthesis was replaced with the higher three homologues [i. e. tetra(ethylene glycol), penta(ethylene glycol), and hexa(ethylene glycol) derivatives] to obtain the MBHA dimers **5b-d**, and their reactivity towards n-butylamine was evaluated in the same conditions used for lower homologue **5a**. The evaluation of the biological properties of the resulting crown ether-paracyclophane hybrid structures revealed a remarkable cytotoxicity in cancer cell lines, providing original new macrocyclic architectures potentially useful as anticancer agents.

## 2. Results and Discussion

### 2.1. Synthesis

**Scheme 2:**
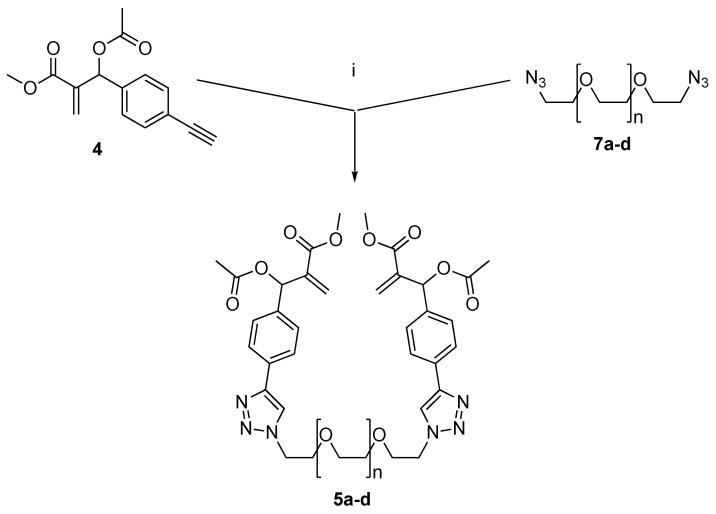
Procedure for the preparation of dimeric MBHA derivatives **5a-d**. Reagents: (i) CuBr(I), DIPEA, CH_3_CN. **5a** and **7a**: n = 1; **5b** and **7b**: n = 2; **5c** and **7c**: n = 3, **5d**, and **7d**: n = 4.

The synthesis of dimeric MBHA derivatives **5b-d** (Scheme 2) was accomplished by the same convergent procedure previously developed for the preparation of lower homologue **5a** [32].

The MBHA component **4** of the convergent synthesis was re-prepared from 4-bromobenzaldehyde as previously described [32]. Similarly, the diazide components **7b-d [37,38,39]** were prepared starting from the commercially available oligo(ethylene glycol) by using the same procedure employed for the preparation of lowest homologue **7a** [32]. The final CuAAC coupling reaction was carried out at room temperature for two hours in acetonitrile in the presence of CuBr(I) as the catalyst and DIPEA as the base to obtain in good yields the oligo(ethylene glycol)-tethered MBHA dimers **5b-d**.

**Scheme 3:**
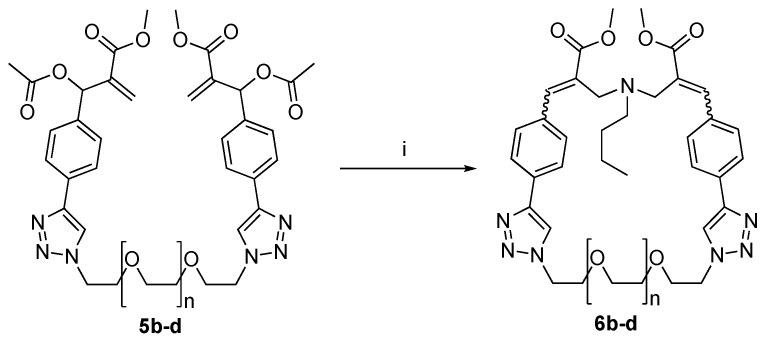
Reaction of oligo(ethylene glycol)-tethered MBHA dimers **5b-d** with n-butylamine. Reagents: (i) CH_3_CH_2_CH_2_CH_2_NH_2_; CHCl_3_.

As already performed with **5a**, compounds **5b-d** were then made to react with n-butylamine (Scheme 3) to obtain the mixtures of macrocyclic diastereomers **6b-d** in good (i.e., 38–62%) yields.

**Figure 2:**
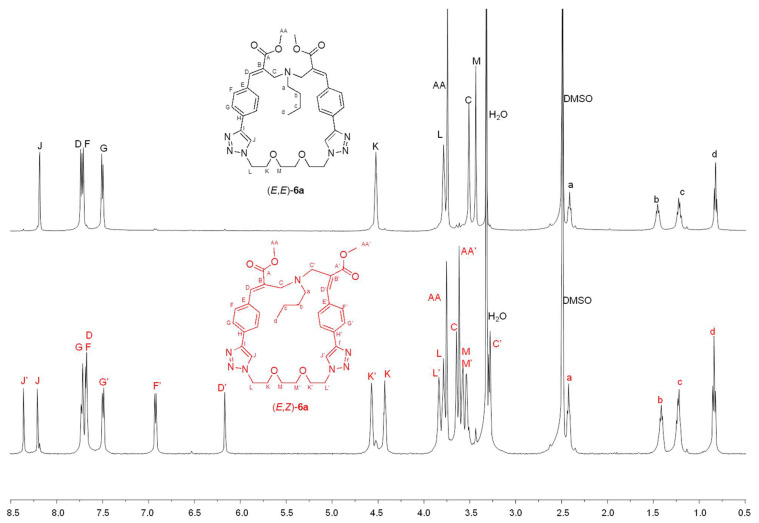
Comparison of the ^1^H NMR spectra recorded (500 MHz, DMSO-d_6_) with crystalline samples of (*E*,*E*)-**6a** and (*E*,*Z*)-**6a**. The signal assignment is reported in red for (*E*,*Z*)-**6a** and in black for (*E*,*E*)-**6a**.

The (*E*,*E*) and (*E*,*Z*) diastereomers of the smallest member **6a** of the series were easily separated by flash chromatography and were obtained in a ratio of 2:1 in the pure crystalline form as white solids [32]. Furthermore, (*E*,*E*)-**6a** was recrystallized from ethyl acetate by slow evaporation, and its structure was confirmed by crystallography [32]. The crystallographic structure of (*E*,*E*)-**6a** was used as the starting point for the structural characterization of the whole set of macrocyclic derivatives **6a-d**. In particular, the ^1^H NMR spectra of (*E*,*E*)-**6a** was assigned and compared with those of the corresponding (*E*,*Z*)-diastereomer (Figure 2) [32].

The comparison of the ^1^H NMR spectra highlights the lack of symmetry of (*E*,*Z*)-**6a** with respect to the symmetric structure of (*E*,*E*)-**6a** as supported by the crystallographic studies [32]. In fact, two distinct sets of signals are visible in almost all the regions of (*E*,*Z*)-**6a** spectra with the exception of the signals of the butylamine moiety. This configurational effect is due to the presence in the unsymmetrical diastereomer molecule of two cinnamic moieties showing opposite E/Z configurations, which could affect also the conformational preferences. It should be noted the great differences in the chemical shift values observed for the signals attributed to protons G’, F’, and D’ with respect to those assigned to the corresponding G, F, and D belonging to the cinnamic moiety in (*E*)-configuration.

**Figure 3:**
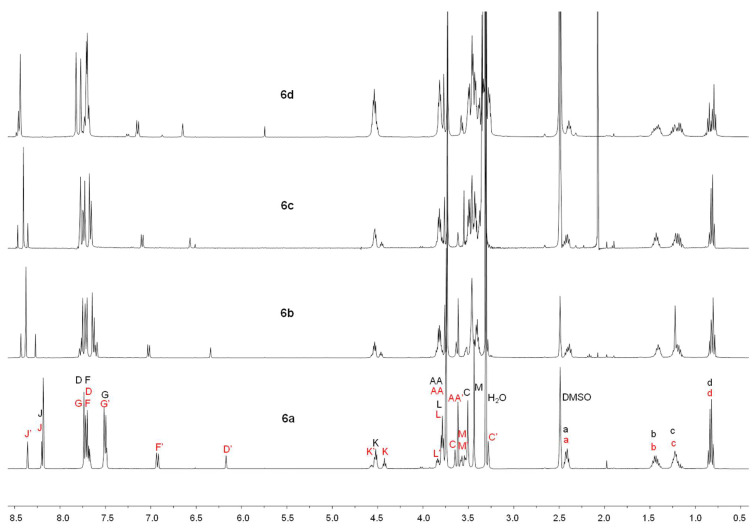
Comparison of the ^1^H NMR spectra recorded (400 MHz, DMSO-d_6_) with the samples obtained from the reaction of dimeric MBHA derivatives **5b-d** with n-butylamine. The ^1^H NMR spectrum in the bottom trace was obtained with the (2:1) mixture of (*E*,*E*)-**6a** (the assignment is reported in black) and (*E*,*Z*)-**6a** (the assignment is reported in red) and is reported as the reference spectrum.

On the other hand, the separation of the diastereomeric mixtures of macrocycle derivatives **6b-d** obtained by the reaction of n-butylamine with MBHA dimers **5b-d** bearing longer oligo(ethylene glycol) tethers was rather difficult, owing to the increasing polarity of the compounds. Thus, ^1^H NMR spectra were performed on the diastereomeric mixtures, and the spectra obtained were compared with the corresponding spectrum obtained with the (2:1) mixture of diastereomers (*E*,*E*)-**6a** and (*E*,*Z*)-**6a** (Figure 3).

**Figure 4:**
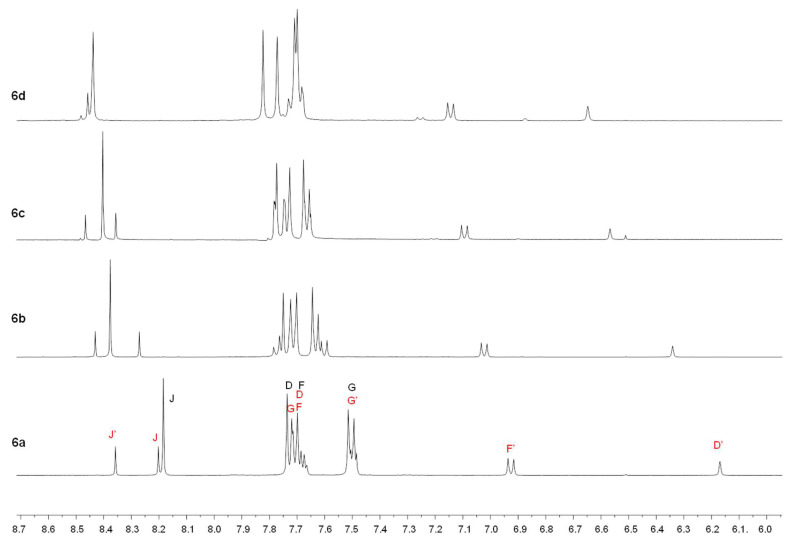
Comparison of the aromatic regions of the ^1^H NMR spectra recorded (400 MHz, DMSO-d_6_) with the samples obtained from the reaction of dimeric MBHA derivatives **5b-d** with n-butylamine. The ^1^H NMR spectrum in the bottom trace was obtained with the (2:1) mixture of (*E*,*E*)-**6a** (the assignment is reported in black) and (*E*,*Z*)-**6a** (the assignment is reported in red) and is reported as the reference spectrum.

The comparison of the spectra shows that the samples were composed by two diastereomers (*E*,*E*) and (*E*,*Z*) of macrocycle derivatives **6b-d** in the ratio of 2:1. These results confirmed that the symmetric (*E*,*E*) diastereomers of **6a-d** were more stable than the corresponding (*E*,*Z*)-diastereomers, and the length of the oligo(ethylene glycol) chain appeared to play a negligible role in affecting the relative stability of the diastereomers. On the other hand, the length of the oligo(ethylene glycol) chain appeared to affect the chemical shift values of the signals in the aromatic regions of ^1^H NMR spectra (Figure 4).

In fact, a progressive down-field shift was observed for all the signals attributed to aromatic protons. This observation appeared to suggest the existence of differences in the relative orientations of the aromatic moieties induced by the macrocycle dimensions.

**Figure 5:**
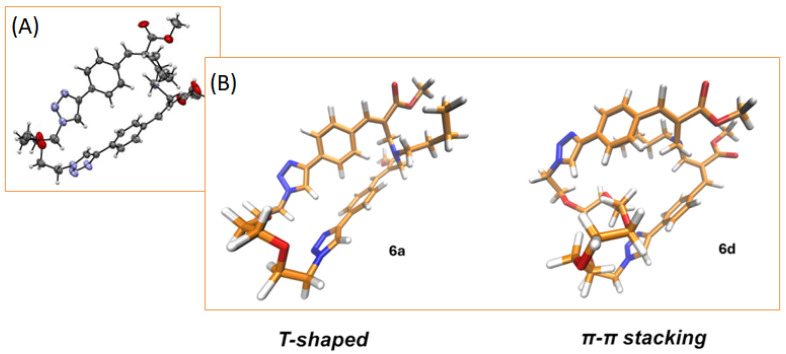
Comparison of (**A**) the X-ray structure of (*E*,*E*)-**6a** [32] with the computed 3D structures (**B**) of (*E*,*E*)-**6a** (**left**) and (*E*,*E*)-**6d** (**right**).

In order to evaluate this assumption, molecular modeling calculations were performed on the symmetric (*E*,*E*) diastereomers of compounds **6a**,**d** that represented the most abundant species in the (2:1) mixtures. In particular, the geometries of the symmetric diastereomers were fully optimized by using density functional theory (DFT) methodology [40] in DMSO as implicit solvent (ε = 46.826). Calculations were performed using B3LYP as functional and 6–31G* as basis set of the GAUSSIAN package (version 16) [41]. The polarized continuum model (PCM) simulating the solvent environment and Grimme dispersion D3 correction for long range (van der Waals) interactions were used [42,43]. The results confirm the differences in the relative orientations of the aromatic moieties induced by the macrocycle dimensions in (*E*,*E*) diastereomers of **6a**,**d** (Figure 5).

In fact, the aromatic moieties of (*E*,*E*)-**6a** were characterized by a compact T-shaped orientation [44] as the one observed in the crystal structure [32], whereas a more relaxed π-π stacked orientation was observed in (*E*,*E*)-**6d** structure. This observation appeared to be fully consistent with the progressive down-field shift in the signal (i.e., the most intense singlet in the aromatic region visible in Figure 4) attributed to the triazole proton (H-J in Figure 2). We assumed that T-shaped interaction of the triazole proton observed in (*E*,*E*)-**6a** structure was capable of shielding the protons and therefore producing an up-field shift with respect to (*E*,*E*)-**6d**. Interestingly, a similar effect was observed also for the aromatic protons (H-G in Figure 2) of the phenyl groups closest to the triazole ring that appeared to share the same orientation of H-J in Figure 5.

Further computational studies are in progress in order to evaluate the conformational features of these interesting macrocyclic derivatives.

### 2.2. Biological Evaluation of Macrocycle Derivatives **6a-d**: In Vitro Cytotoxicity Assay

**Figure 6:**
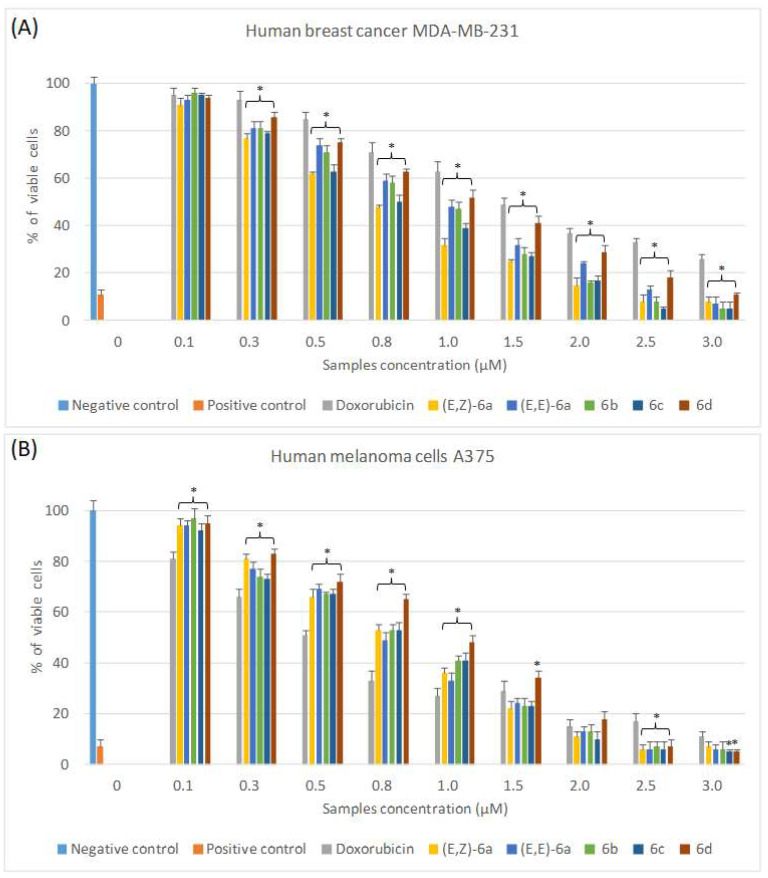
Percentage of viable (**A**) human breast cancer MDA-MB-231 and (**B**) human melanoma cells A375 after 24 h of contact with different concentrations of test compounds, as determined by the Neutral Red Uptake. Negative control: complete medium; positive control: 4 µL of Lysis Solution (a 1:250 dilution of 9% wt:vol Triton X-100) per 100 μL of cells in culture medium. Data are mean ± SD of three experiments run in six replicates. * Values are statistically different versus Doxorubicin, *p* < 0.05.

Non-confluent, adhered human breast cancer MDA-MB-231 and human melanoma A375 cells were incubated with increasing concentrations of (*E*,*Z*)-**6a**, (*E*,*E*)-**6a**, **6b**, **6c**, **6d**, and Doxorubicin (used as positive control) [45], ranging from 0.1 to 3.0 µM. Cells were analyzed after 24 h, and the results (Figure 6) showed that both exponentially growing human tumor cell lines exhibited characteristic dose–response trends for all six tested compounds [46,47].

In particular, as reported in Figure 6A, the cytotoxic effect of all tested samples towards MDA-MB-231 cells increased with increasing concentrations, but to varying degrees [48,49]. Doxorubicin and compound **6d** demonstrated a lower cytotoxic effect compared to (*E*,*Z*)-**6a**, (*E*,*E*)-**6a**, **6b**, and **6c** at concentrations ≥ 0.3 µM. Compounds (*E*,*Z*)-**6a** and **6c** were the most cytotoxic at concentrations ranging from 0.3 to 1.5 µM; (*E*,*E*)-**6a** and **6b** were more cytotoxic than Doxorubicin but less than (*E*,*Z*)-**6a** and **6c** from 0.3 to 2.5 µM.

The IC_50_ value for MDA-MB-231 cells was 1.5 µM for Doxorubicin, 1.0 µM for (*E*,*E*)-**6a**, **6b**, **6d**, and 0.8 µM for both (*E*,*Z*)-**6a** and **6c** (Table 1).

Similarly to MDA-MB-231 cells, the cytotoxic effect of all tested samples towards human melanoma A375 cells increased with increasing concentrations, but to varying degrees [50]. As reported in Figure 6B, at concentrations ranging from 0.1 to 1.0 µM Doxorubicin demonstrated a higher cytotoxic effect compared to (*E*,*Z*)-**6a**, (*E*,*E*)-**6a**, **6b**, and **6c** towards human melanoma A375 cells, while, at concentrations higher than 1.5 µM, the latter was found to be more cytotoxic. The least cytotoxic sample was compound **6d**, which showed minimal ability to interfere with cell viability at concentrations ranging from 0.3 to 2.0 µM. No significant differences were found among the other tested compounds, (*E*,*Z*)-**6a**, (*E*,*E*)-**6a**, **6b**, and **6c**.

This trend was also demonstrated by the IC_50_ values for A375 cells: 0.5 µM for Doxorubicin (the most cytotoxic compound), 1.0 µM for sample **6d** (the least cytotoxic), and 0.8 µM for (*E*,*Z*)-**6a**, (*E*,*E*)-**6a**, **6b**, and **6c**, which all showed the same degree of cytotoxicity.

Overall, the slight differences (see Table 1) observed in the cytotoxicity potency of macrocyclic derivatives **6a-d** led us to assume that the cytotoxicity mechanism could be linked to a structurally non-specific interaction of the macrocyclic compounds with some key cellular components such as membrane. This assumption appeared to be in full agreement with the current literature. In fact, evidence has been reported that crown ethers are capable of inhibiting tumor-cell growth through the disruption of potassium ion homeostasis leading to cell cycle perturbations and apoptosis [13]. On the other hand, an alternative hypothesis is that the interaction of macrocyclic derivatives **6a-d** with cellular membranes could lead to loss of membrane integrity [51]. However, specifically designed experiments are required in order to gain experimental evidence supporting this hypothesis.

## 3. Conclusions

In the aim of obtaining a small series of new macrocyclic crown ether-paracyclophane hybrid architectures, a Morita–Baylis–Hillman acetate bearing a clickable phenylactylene moiety was dimerized by click-chemistry CuAAC reaction employing oligo(ethylene glycol) diazide derivatives with the formation of a small series of dimeric MBHA. The reaction of these dimeric MBHA derivatives with n-butylamine afforded the designed macrocyclic crown ether-paracyclophane hybrid architectures, which were evaluated for their potential cytotoxicity in human breast cancer MDA-MB-231 and human melanoma A375 cells. Very interestingly, tested compounds (*E*,*Z*)-**6a**, (*E*,*E*)-**6a**, **6b-d** showed remarkable cytotoxicity with IC_50_ values comparable with reference anticancer agent Doxorubicin. Thus, this exploration approach provides original new macrocyclic architectures potentially useful as anticancer agents. The versatility of our synthetic approach can rapidly lead to the expansion of this family of compounds by exploiting different nucleophilic agents, such as amino acids or peptides, often exploited in anticancer drugs to increase cell penetration and cell targeting [52,53,54,55,56,57,58,59,60].

## 4. Experimental Section

### 4.1. Synthesis

Column chromatography was carried out using a Merck (Darmstadt, Germany) silica gel 60 (230–400 mesh). Merck TLC plates, silica gel 60 F_254_ were used for TLC. NMR spectra were recorded with a Bruker DRX-500 AVANCE (Billerica, MA) or a Bruker DRX-400 AVANCE III spectrometer in the indicated deuterated solvents (TMS as internal standard): the values of the chemical shifts are expressed in ppm and the coupling constants (*J*) in Hz. Mass spectrometry experiments were carried out using an Agilent (Santa Clara, CA) 1100 LC/MSD operating with an electrospray source.

General procedure for the synthesis of compounds **5b-d**.

To a mixture of **4** (2 equivalents) and the appropriate diazide derivative **7b-d** (1 equivalent) in anhydrous acetonitrile, DIPEA (0.75 equivalents) and CuBr(I) (0.75 equivalents) were added in the sequence. The resulting mixture was stirred at r.t. under a nitrogen atmosphere for 2 h. Then, the reaction mixture was concentrated under reduced pressure, and the resulting residue was dissolved in dichloromethane and washed with NH_4_OH. The organic phase was dried over Na_2_SO_4_ and concentrated under reduced pressure. The purification of the residue by flash chromatography with the indicated eluent furnished the desired MBHA dimer **5b-d**.

Dimethyl 2,2′-((((((oxybis(ethane-2,1-diyl))bis(oxy))bis(ethane-2,1-diyl))bis(1*H*-1,2,3-triazole-1,4-diyl))bis(4,1-phenylene))bis(acetoxymethylene))diacrylate (**5b**).

The title compound was obtained starting from **7b** (57 mg, 0.23 mmol) as a thick light-yellow oil (66 mg, yield 37%) using ethyl acetate-methanol 95:5 *v*/*v* as the eluent. ^1^H NMR (400 MHz, CDCl_3_): 2.11 (s, 6H), 3.53 (s, 8H), 3.70 (s, 6H), 3.80 (t, *J* = 5.0, 4H), 4.49 (t, *J* = 5.0, 4H), 5.90 (s, 2H), 6.40 (s, 2H), 6.67 (s, 2H), 7.42 (d, *J* = 8.2, 4H), 7.78 (d, *J* = 8.2, 4H), 7.89 (s, 2H). MS (ESI): *m*/*z* 783.3 [M + Na^+^].

Dimethyl 2,2′-((((3,6,9,12-tetraoxatetradecane-1,14-diyl)bis(1*H*-1,2,3-triazole-1,4-diyl))bis(4,1-phenylene))bis(acetoxymethylene))diacrylate (**5c**).

The title compound was obtained starting from **7c** (83.5 mg, 0.29 mmol) as a thick yellow oil (0.123 mg, yield 53%) using ethyl acetate-methanol 95:5 *v*/*v* as the eluent. ^1^H NMR (400 MHz, CDCl_3_): 2.11 (s, 6H), 3.46–3.62 (m, 12H), 3.70 (s, 6H), 3.87 (t, *J* = 4.9, 4H), 4.57 (t, *J* = 4.9, 4H), 5.90 (s, 2H), 6.41 (s, 2H), 6.67 (s, 2H), 7.45 (d, *J* = 8.2, 4H), 7.89 (d, *J* = 8.2, 4H), 8.15 (s, 2H). MS (ESI): *m*/*z* 827.3 [M + Na^+^].

Dimethyl 2,2′-((((3,6,9,12,15-pentaoxaheptadecane-1,17-diyl)bis(1*H*-1,2,3-triazole-1,4-diyl))bis(4,1-phenylene))bis(acetoxymethylene))diacrylate (**5d**).

The title compound was obtained starting from **7d** (87 mg, 0.26 mmol) as a pale-yellow oil (0.137 mg, yield 62%) using ethyl acetate-methanol 95:5 *v*/*v* as the eluent. ^1^H NMR (400 MHz, CDCl_3_): 2.10 (s, 6H), 3.56 (m, 16H), 3.69 (s, 6H), 3.87 (t, *J* = 4.9, 4H), 4.55 (t, *J* = 4.9, 4H), 5.88 (s, 2H), 6.40 (s, 2H), 6.68 (s, 2H), 7.41 (d, *J* = 8.1, 4H), 7.80 (d, *J* = 8.1, 4H), 7.96 (s, 2H). MS (ESI): *m*/*z* 871.3 [M + Na^+^].

Reaction of homodimer **5b** with n-butylamine.

Dimethyl (1^4^*Z*,11^4^*Z*)-6-butyl-1^1^*H*,11^1^*H*-14,17,20-trioxa-6-aza-1,11(4,1)-ditriazola-2,10(1,4)-dibenzenacyclodocosaphane-3,8-diene-4,8-dicarboxylate (**6b**).

Compound **5b** (66 mg, 0.087 mmol) was solubilized in CHCl_3_ (11 mL), and then, n-butylamine (17 µL, 0.174 mmol) was added. The resulting mixture was refluxed for 24 h. Subsequently, this mixture was concentrated under reduced pressure and the organic residue obtained was purified by flash chromatography with ethyl acetate-methanol (8:2) as the eluent to obtain **6b** as white solid (35.5 mg, yield 57%). The ^1^H NMR spectrum showed the presence of (*E*,*E*) and (*E*,*Z*) diastereomers as a (2:1) mixture (Figure 3 and Figure 4). MS (ESI): *m*/*z* 714.3 [M + H^+^]. MS (ESI): *m*/*z* 736.3 [M + Na^+^].

Reaction of homodimer **5c** with n-butylamine.

Dimethyl (1^4^*Z*,11^4^*Z*)-6-butyl-1^1^*H*,11^1^*H*-14,17,20,23-tetraoxa-6-aza-1,11(4,1)-ditriazola-2,10(1,4)-dibenzenacyclopentacosaphane-3,8-diene-4,8-dicarboxylate (**6c**).

Compound **5c** (0.114 g, 0.14 mmol) was solubilized in CHCl_3_ (17 mL), and then, n-butylamine (28 µL, 0.28 mmol) was added. The resulting mixture was refluxed for 24 h. Subsequently, this mixture was concentrated under reduced pressure, and the organic residue obtained was purified by flash chromatography with ethyl acetate-methanol (85:15) as the eluent to obtain **6c** as waxy white solid (66 mg, yield 62%). The ^1^H NMR spectrum showed the presence of (*E*,*E*) and (*E*,*Z*) diastereomers as a (2:1) mixture (Figure 3 and Figure 4). MS (ESI): *m*/*z* 758.3 [M + H^+^]. MS (ESI): *m*/*z* 780.3 [M + Na^+^].

Reaction of homodimer **5d** with n-butylamine.

Dimethyl (1^4^*Z*,11^4^*Z*)-6-butyl-1^1^*H*,11^1^*H*-14,17,20,23,26-pentaoxa-6-aza-1,11(4,1)-ditriazola-2,10(1,4)-dibenzenacyclooctacosaphane-3,8-diene-4,8-dicarboxylate (**6d**).

Compound **5d** (0.137 g, 0.16 mmol) was solubilized in CHCl_3_ (20 mL) and then n-butylamine (32 µL, 0.32 mmol) was added. The resulting mixture was refluxed for 24 h. Subsequently, this mixture was concentrated under reduced pressure. The organic residue obtained was purified by flash chromatography with ethyl acetate-methanol (9:1) as the eluent to obtain **6d** as yellow waxy oil (49 mg, yield 38%). The ^1^H NMR spectrum showed the presence of (*E*,*E*) and (*E*,*Z*) diastereomers as a (2:1) mixture (Figure 3 and Figure 4). MS (ESI): *m*/*z* 802.4 [M + H^+^]. MS (ESI): *m*/*z* 824.4 [M + Na^+^].

### 4.2. Biological Evaluation of Macrocycle Derivatives **6a-d**

#### 4.2.1. Materials

Dulbecco’s Modified Eagle’s Medium, trypsin solution, and all the solvents used for cell culture were purchased from Merck. Human breast cancer MDA-MB-231 cells and human melanoma cells A375 were purchased from American Type Culture Collection (Manassas, VA, USA).

#### 4.2.2. In Vitro Cytotoxicity Assay

Both human breast cancer MDA-MB-231 and human melanoma cells A375 cells were maintained in DMEM at 37 °C in a humidified atmosphere containing 5% CO_2_. The culture medium was supplemented with 10% fetal calf serum (FCS), 1% L-glutamine-penicillin-streptomycin solution, and 1% MEM Non-Essential Amino Acid Solution. Once at confluence, cells were washed with PBS 0.1M, detached with trypsin-EDTA solution, and then centrifuged at 1000 rpm for 5 min. The pellet was re-suspended in the medium solution (dilution 1:15).

Cells (1.5 × 10^4^) suspended in 1 mL of complete medium were seeded in each well of a 24 well round multidish and incubated at 37 °C in an atmosphere of 5% CO_2_. Once reached 50% of confluence (i.e., after 24 h of culture), the culture medium was discharged and the test compounds, properly diluted in completed medium, were added to each well.

The stock solutions of test compounds (*E*,*Z*)-**6a**, (*E*,*E*)-**6a**, **6b**, **6c**, **6d**, and Doxorubicin were prepared in DMSO. The following concentrations of each sample were tested: 0.1; 0.3; 0.5; 0.8; 1.0; 1.5; 2.0; 2.5; 3.0 µM.

Each experiment was repeated three times, and all samples were set up in six replicates. Complete medium was used as negative control and 4 µL of Lysis Solution (a 1:250 dilution of 9% wt:vol Triton X-100) per 100 μL of cells in culture medium, as positive control [61]. After 24 h of incubation, cell viability was evaluated by Neutral Red uptake, as previously reported [62].

#### 4.2.3. Statistical Analysis

Multiple comparison was performed by one-way ANOVA using GraphPad version 10.4 [63]. Differences with *p* < 0.05 were considered significant.

## Figures and Tables

**Table 1:** IC_50_ value (µM) of the tested samples towards MDA-MB-231 and A375IC50 cells.

Compounds	IC_50_ (µM)	
	MDA-MB-231 Cells	A375 Cells
Doxorubicin	1.5	0.5
(*E*,*Z*)-**6a**	0.8	0.8
(*E*,*E*)-**6a**	1.0	0.8
**6b**	1.0	0.8
**6c**	0.8	0.8
**6d**	1.0	1.0

## Data Availability

The data presented in this study are contained within the article. Requests for further details can be directed to the corresponding author.
